# Disclosing possible nonmedically indicated cesarean sections in 5 high-volume urban maternity units in Tanzania: a criterion-based clinical audit

**DOI:** 10.1016/j.xagr.2024.100437

**Published:** 2024-12-21

**Authors:** Sarah Hansen, Monica Lauridsen Kujabi, Rikke Damkjær Maimburg, Anna Macha, Luzango Maembe, Idrissa Kabanda, Manyanga Hudson, Rukia Juma Msumi, Mtingele Sangalala, Natasha Housseine, Brenda Sequeira D'mello, Kidanto Hussein, Thomas van den Akker, Dan Wolf Meyrowitsch, Nanna Maaløe

**Affiliations:** a Department of Clinical Medicine, Occupational Medicine, Aarhus University, Aarhus, Denmark (Hansen and Maimburg); b Global Health Section, Department of Public Health, University of Copenhagen, Copenhagen, Denmark (Kujabi, D'mello, Meyrowitsch, and Maaløe); c Department of Gynecology and Obstetrics, Aarhus University Hospital, Aarhus, Denmark (Kujabi); d Department of Midwifery, University College of Northern Denmark, Hjoerring, Denmark (Maimburg); e Medical College East Africa, Aga Khan University, Dar es Salaam, Tanzania (Macha, Housseine); f Department of Gynecology and Obstetrics, Mwanyanamala Regional Referral Hospital, Dar es Salaam, Tanzania (Maembe); g Department of Gynecology and Obstetrics, Sinza Regional Hospital, Dar es Salaam, Tanzania (Kabanda); h Department of Gynecology and Obstetrics, Temeke Regional Referral Hospital, Dar es Salaam, Tanzania (Hudson); i Department of Gynecology and Obstetrics, Rangi Tatu Maternity Hospital, Dar es Salaam, Tanzania (Msumi); j Department of Gynecology and Obstetrics, Amana Regional Referral Hospital, Dar es Salaam, Tanzania (Sangalala); k Comprehensive Community Based Rehabilitation in Tanzania, Dar es Salaam, Tanzania (D'mello); l Athena Institute, VU University Amsterdam, Amsterdam, the Netherlands (Akker); m Department of Obstetrics and Gynecology, Leiden University Medical Centre, Leiden, the Netherlands (Akker); n Department of Gynecology and Obstetrics, Copenhagen University Hospital –Hvidovre Hospital, Copenhagen, Denmark (Maaløe)

**Keywords:** low-income countries, sub-Saharan Africa, Tanzania, PartoMa, sub-standard care, urban disadvantage, trial of labor, fetal distress

## Abstract

**Background:**

Globally, the cesarean section rate has increased dramatically with many cesarean sections being performed on questionable medical indications. Particularly in urban areas of sub-Saharan Africa, the cesarean section rate is currently increasing rapidly. This potentially undermines the positive momentum of increased facility births and may be a central contributor to a growing "urban disadvantage" in maternal and perinatal health, which is seen in some settings.

**Objective:**

To assess to what extent cesarean section indications follow evidence-based, locally co-created audit criteria in five urban, high-volume maternity units in Dar es Salaam, Tanzania, and identify reasons contributing to nonmedically indicated cesarean sections.

**Study Design:**

This was a retrospective cross-sectional study conducted, from October 1st, 2021 to August 31st, 2022. A criterion-based audit with pre-defined, localized audit criteria was used to examine the clinical case-files of all women who gave birth by cesarean section during 3-month periods at the 5 maternity units. Primary outcomes were the cesarean section rate, indications for cesarean section, and proportion of nonmedically indicated cesarean sections. The PartoMa study is registered in ClinicalTrials.gov (NCT04685668).

**Results:**

Overall, the cesarean section rate was 31.5% (2949/9364), of which 2674/2949 (90.7%) cesarean sections had available data for analysis. Main indications were previous cesarean section (1133/2674; 42.4%), prolonged labor (746/2674; 27.9%), and fetal distress (554/2674; 20.7%). Overall, 1061/2674 (39.7%) did not comply with audit criteria at the time cesarean section was decided. Main reasons were one previous cesarean section with no trial of labor (526/1061; 49.6%); reported prolonged labor without actual slow progress (243/1061; 22.9%); and fetal distress with normal fetal heart rate at time of decision (211/1061; 19.9%).

**Conclusion:**

Two in 5 cesarean sections were categorized as nonmedically indicated at time of decision. Particularly, fear of poor outcomes and delay in accessing emergency surgery may cause resource-consuming "defensive decision-making" for cesarean section. Investments in conducive urban maternity units are crucial to ensure safe vaginal births and to reach a population-based approach that provides best possible timely care for all with the limited resources available.

**AJOG Global Reports at a Glance**

**Why was this study conducted?**

Urban Tanzania has experienced a rapid rise in cesarean sections. There has been little attention to understand clinical management and decision-making preceding cesarean sections in these urban settings.

**Key findings**

In the 5 highest-volume maternity units in Dar es Salaam, 39.7% of cesarean sections were deemed nonmedically indicated, based on predefined, localized criteria. Main reasons included absence of trial of labor after one previous cesarean section and nonsubstantiated diagnoses of prolonged labor and fetal distress.

**What does this study add to what is known?**

With more than half of global births now occurring in urban areas, this study provides insight into drivers of rapidly increasing cesarean section rates in Dar es Salaam. It highlights the need for context-specific decision-support tools on mode of birth, alongside urban investments to create conducive maternity units that prevent both over- and undertreatment of women.

## Introduction

Globally, the cesarean section (CS) rate has increased dramatically, now reaching 21.1% and forecasted to be 28.5% in 2030.^1^ CSs remain crucial in obstetrics to save lives, and some women still lack access to timely CS.^2^ Yet, the growing “CS pandemic” is dominated by CSs being performed on questionable medical indications.^3^^,^^4^^,^^5^ This results in preventable mortality and morbidity that threaten to counteract hard-won improvements in maternal and perinatal health.^2^^,^^6^

The overuse of CSs is of particular concern in sub-Saharan Africa where associated risks are highest: 1% of women and 8% of babies are estimated to die during or after CS, which is 100- and 50-fold the rates in high-income countries.^6^ Furthermore, in subsequent pregnancies, a previous CS predisposes to repeated CS, uterine rupture and abnormal placentation.^7^, ^8^, ^9^ While national rates of CS in sub-Saharan Africa remain lower than in other world regions, the urban rates are increasing rapidly, which may contribute to a growing trend towards an "urban disadvantage" in maternal and perinatal health.^1^^,^^10^ Moreover, this rapid urban growth, with urban poverty, inadequate infrastructure, and congested maternity facilities, challenges the traditional rural-centric approach in maternal and newborn health programs, highlighting the need for a shift in health strategies.^11^, ^12^, ^13^ Notably, the speed of urban growth in sub-Saharan Africa is forecasted to accelerate further, with the urban population expected to reach 60% by 2050.^14^

Tanzania exemplifies the urban challenges in providing timely childbirth care. Since 2016, maternal and perinatal mortality have been significantly higher in urban Tanzania than in the country's rural areas.^15^, ^16^, ^17^ This is despite 76% of urban women attending at least 4 antenatal visits and 94% giving birth in facilities, indicating that the quality of care often falls short, leading to a dangerous co-existence of “too little, too late” and “too much, too soon” care.^2^^,^^16^

In Tanzania's biggest city, Dar es Salaam (DSM), which is among the world's fastest-growing cities, the population-based CS rate has increased from 17% in 2016 to 26% in 2022.^15^^,^^16^^,^^18^ Here, we present an in-depth, criteria-based audit of the quality of clinical management and decision-making preceding CSs in 5 of the city's highest volume maternity units. We thereby aim to assess to what extent CS indications follow evidence-based, locally co-created audit criteria, and identify reasons contributing to nonmedically indicated CSs.

## Materials and Methods

### Study design and method of analysis

**Figure 1:**
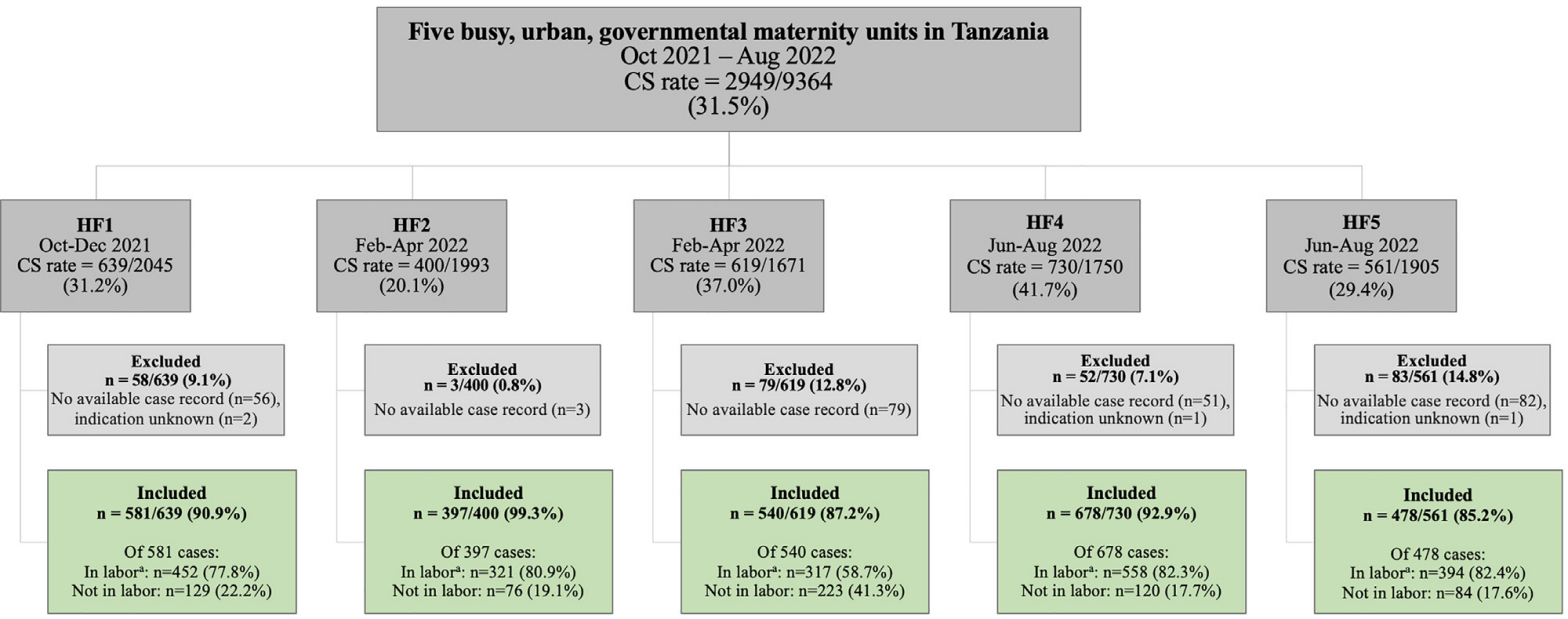
The facility-based CS rates and the total number of women included in the study. ^a^If unknown status of labor on admission, women were defined to be in labor.

A retrospective cross-sectional observational study was performed using a criterion-based audit to examine the clinical case-files of all women who gave birth by CS during a 3-month period at each of 5 high-volume maternity units in DSM, Tanzania.^19^^,^^20^ Data collection took place between October, 1st 2021 and August, 31st 2022, as part of a baseline situational analysis for the PartoMa Birth scale-up study (Figure 1).^21^^,^^22^ Data on clinical management preceding the decision to perform CS were collected and compared to preselected, localized criteria of best possible care (Table 1).^23^ Reporting of the study follows the STROBE guidelines.^24^

Hansen. Disclosing possible nonmedically indicated cesarean sections in 5 high-volume urban maternity units in Tanzania: a criterion-based clinical audit. AJOG Glob Rep 2024.

**Table 1:** Evidence-based and locally agreed audit criteria grouping indications for CS into “medically indicated”, “nonmedically indicated” and “unclear if medically indicated”^23^

Medically indicated CS (i.e. absolute indications for CS)	Nonmedically indicated CS (i.e. indications that do not require CS)	Unclear if medically indicated CS (i.e. nonassessable indications due to suboptimal recording in case-files)
- **Abnormal presentation:** Brow presentation, compound presentation, face mentoposterior, footling breech, oblique lie, transverse lie, shoulder presentation, twins if first is breech - **Abruptio placenta** **- Breech**^a^: Nulliparous or failed trial of labor in multiparous - **Cord prolapse** with pulsating cord - **Fetal distress:** Abnormal FHR at time of decision (<100;>180 bpm) - **One previous CS:** Failed trial of labor - **Placenta praevia** **- Previous reconstructive vaginal surgery** (fistula repair etc.) - **Prolonged labor/Cephalopelvic disproportion** **- Retained second twin** **- Severe hypertensive disorders** in pregnancy with another absolute or nonassessable indication^b^ - **Two or more previous CS** **- Uterine rupture** incl. severe scar pain/impending uterine rupture - **Vacuum extraction failure** **- Other absolute indications**^c^	- **One previous CS**^d^: No trial of labor^e^ - **Prolonged labor/Cephalopelvic disproportion**^f^**:** **-** CS in latent labor (cervical dilatation <4 cm) lasting less than 2 days (unless grossly abnormal pelvis) - CS during first stage, active labor (cervical dilatation 4-<10 cm) before crossing the partograph's action line - CS during second stage labor where duration of second stage is less than 2 hours for multiparous women and less than 3 hours for nulliparous women (unless failed vacuum extraction or progressive signs of obstruction described) g - **Fetal distress**^h^: Normal FHR at time of decision (120-160 bpm) - **Hypertensive disorders in pregnancy:** Mild-moderate hypertensive disorders or severe hypertensive disorders as only indication^b^ - **Breech**^a^**:** No trial of labor in multiparous with complete or frank breech^e^ - **Other indications that alone do not require a CS**^i^	- **Fetal distress**: Borderline FHR (100-119;161-180 bpm) or no FHR documented at time of decision - **Previous CS**: Unknown number of previous CS - CS with missing information to categorize whether “nonmedically indicated” or “medically indicated”^d^^/^e^/^j

If presentation was not written in the case-file it was assumed to be cephalic.

CS, Cesarean section; FHR, Fetal heart rate

a Medically indicated (absolute): Breech presentation with previous CS or fetal weight >4 kg

b Severe hypertensive disorders: Severe pre-eclampsia or eclampsia documented in the case-file. Mild hypertensive disorders: Hypertension or pre-eclampsia documented in the case-file

c Other absolute indications: Bartholin's edema, cervical/uterine prolapse, cervical stenosis, hip dislocation in previous labor, obstructive tumor, pelvic injury, psychosis, transverse vaginal septum

d Medically indicated (absolute): One previous CS with noncephalic presentation or multiple gestation

e Trial of labor is defined as being in labor for a minimum of 4 hours. If unknown status of labor on admission, women were defined to be in labor.

f Unclear if medically indicated: Referred women with cervical dilatation >6 cm upon admission or where referral diagnosis was prolonged labor, breech pregnancies, multiple pregnancies, intrauterine fetal deaths, failed induction, and women with a previous CS.

g Definition of progressive signs of obstruction: Head is >1/5 palpable above the pelvic brim per abdomen, station at level of ischial spine or above, or severe caput and molding (+3)).

h Definition of fetal distress: Fetal distress, nonreassuring fetal status and reduced-fetal movement documented as indication for CS

i Other indications that alone do not require a CS: Age below 16 years, bad obstetric history, chorioamnionitis (no fetal distress/shock/prolonged labor), elderly maternal age, endometriosis, grand multiparity, intrauterine contraceptive device, intrauterine fetal death, long interpregnancy interval, maternal distress, oligohydramnios with normal FHR, placenta calcification, polyhydramnios, post date, precious baby, preterm premature rupture of membranes, premature rupture of membranes, twins (not conjoined, no malpresentation)

j Unclear if medically indicated: Anemia, antepartum bleeding (no placenta abruptio, no placenta praevia), failure of induction, fetal malformation, genital warts/vaginal infection, human immunodeficiency virus, intrauterine growth restriction, prolonged premature rupture of membranes, uterine myoma.

The audit criteria were based on the locally co-created PartoMa Birth Clinical Practice Guidelines, informed by surveys of women's childbirth experiences, influenced by qualitative observations in the health facilities (HF) and co-created with frontline healthcare providers to create realistic, safe and evidence-based guidance on intrapartum care.^23^^,^^25^ The Clinical Practice Guidelines are based on national and international evidence-based guidelines, externally peer-reviewed, and approved by the Regional health authorities for use in the included facilities.^21^ Consequently, the aim was for the audit criteria to reflect the best possible clinical practice when deciding on CS in the audited clinical contexts. Similar audit criteria have been applied successfully in comparable low-resource settings.^4^^,^^26^, ^27^, ^28^, ^29^

### Study setting

The 5 study HF, all government-owned maternity units in DSM, provide comprehensive obstetric and neonatal care. Two are Municipal Maternity Hospitals (HF1 and HF2), and 3 are regional referral hospitals (HF3, HF4, and HF5), predominantly serving women of lower socioeconomic status (Figure S1). These hospitals’ maternity units have been the most congested in DSM for more than a decade, with each now providing services for 7,000 to 14,000 births annually. Due to staff shortage, nurse-midwives attend 1 to 7 laboring women simultaneously.

Monitoring devices for maternal vital signs and fetal heart rate (FHR) are limited in number, and analgesia during labor is unavailable. Occasionally, the HF face shortages of other supplies, such as essential medication (e.g., antihypertensives), blood products and vacuum extractors. Furthermore, during data collection, the hospitals lacked clinical guidelines regarding intrapartum management and when to perform CS. Labor is monitored using the partograph, and FHR monitoring is usually assessed using a Pinard fetoscope, occasionally supplemented by a fetal doppler.

The maternity units are led by 1 or 2 obstetricians and a nurse-in-charge, with births attended by nurse-midwives and occasionally by medical doctors or clinical officers. Each facility has 1 operating theatre for obstetric emergencies and planned surgeries, where CS is performed by a medical doctor or clinical officer with no financial incentives linked to the procedure. Intravenous antibiotics are routinely administered to women undergoing CS. Neonatal high-care units are led by pediatricians in the referral hospitals, while sick newborns from HF1 and HF2 are referred there.

### Data collection and management

All women who gave birth by CS at the 5 study sites during the 3 study months were included. Women who gave birth by CS were identified through each facility's birth register and cross-checked with the hospitals’ surgical registers. Case-files were retrieved within one month after childbirth. Women with an inaccessible case-file or those lacking recorded indications for CS, were excluded from analysis.

Data for the included women were extracted from case-files, partographs, birth registers and operating room records. The data were recorded in pretested case extraction forms using KoBoToolbox.^30^ Data collection was done by SH and 3 research assistants, all with a medical background. Prior to data collection, data collectors received training to ensure alignment in the interpretation of case-files. In case of ambiguous documentation, staff members from the labor ward were consulted. To maintain data retrieval quality, 810/2674 (30.3%) of files were double-entered; all discrepancies were corrected, and data collectors received continuous training to prevent further errors. In case of discrepancies, MLK or NM served as a tie breaker. If a procedure was not documented, it was assumed that it had not been performed.^31^

### Variables

The audit form included background characteristics, intrapartum care, crucial time-points (e.g., admission, decision on CS, birth), status of labor at time of CS decision, and perinatal and maternal outcomes. Sociodemographic indicators (e.g. economic and educational status) were not available.

Moreover, audit criteria were selected to categorize CS indications into: 1) medically indicated; 2) nonmedically indicated; and 3) unclear if medically indicated (Table 1). Some CSs had more than one indication. The group *medically indicated* included CSs where at least one indication was considered medically indicated. The category *nonmedically indicated* included indications that, according to the audit criteria, did not require a CS. The category *unclear if medically indicated* included CSs with incomplete case-files. As cases of CS due to prolonged labor needed more complex analysis, these are reported elsewhere.^32^

We included all CS indications documented in the managing clinician's and surgeon's notes regarding the CS decision. The following were grouped as "prolonged labor": "poor progress of labor," "failure of augmentation," "cervical arrest," "cervical dystocia," "cephalopelvic disproportion," "obstructed labor," and "big baby." The following indications were grouped as fetal distress: "fetal distress," "non-reassuring fetal status", "reduced fetal kicks/movement" and "meconium-stained liquor". Women with unknown labor progress upon admission were categorized as having been in labor.

For case-files without documented time of birth, this time was estimated by deducting 45 minutes from the time of reaching the postpartum ward after surgery, based on hospital staff feedback.

### Statistical analyses

**Figure 2:**
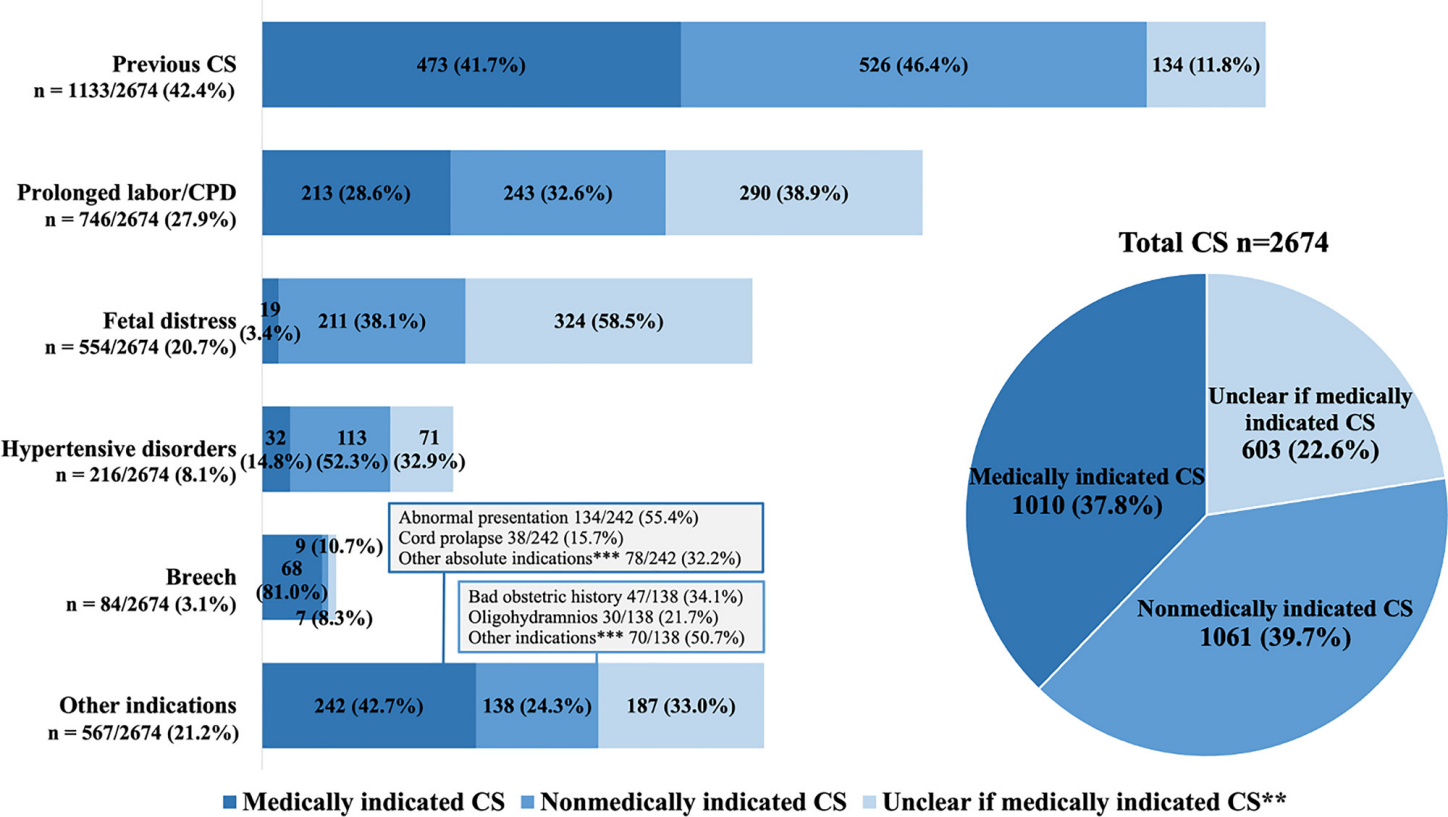
Distribution of indications for CS and an overview of “medically indicated CS”, “nonmedically indicated CS” and “unclear if medically indicated CS”.* Each case may have more than one indication. * If more than one indication for CS, both indications were reviewed. The group “medically indicated CS” includes CSs where at least one indication was considered absolute, for example, placenta praevia. The group “nonmedically indicated CS” includes indications that, according to the criteria, do not require a CS. The group “unclear if medically indicated” includes CSs that could not be assessed. Within each of the commonest indications, “medically indicated CS” is defined as the indication being in accordance with audit criteria (absolute indication). “Nonmedically indicated CS” is defined as the indication not being in accordance with audit criteria, and no other absolute or nonassessable indication is present. “Unclear if medically indicated” is defined as the indication being either nonassessable or not in accordance with audit criteria, yet another absolute or nonassessable indication for CS is present. ** The proportion in “Unclear if medically indicated” that is nonmedically indicated due to another absolute indication: Previous CS 7/134 (5.2%), prolonged labor/CPD 64/290 (22.1%), fetal distress 67/324 (20.7%), hypertensive disorders in pregnancy 29/71 (40.8%), breech 2/9 (22.2%) and other indications 41/187 (22.0%). *** Other absolute indications in "medically indicated CS": Placenta abruptio n=23, placenta praevia n = 23, uterine rupture n = 7, retained twin n = 5, vacuum failure n = 5, previous reconstructive vaginal surgery n = 4, cervical stenosis n = 3, obstetric tumor n = 3, cervical prolapse n = 2, bartholin's edema n = 1, hip dislocation in previous pregnancy n = 1, pelvic injury n = 1, psychosis n = 1, transverse vaginal septum n = 1. Other indications in “nonmedically indicated CS”: Placenta calcification n = 17, premature rupture of membranes n = 14, long interpregnancy interval n = 12, post date n = 10, grand multiparity n = 5, elderly maternal age n = 4 chorionamnioitis n = 3, polyhydramnios n = 3, twins n = 3, precious baby n = 2, intrauterine fetal death n = 2, maternal distress n = 2, below 16 years of age n = 1, endometriosis n = 1. CPD, Cephalopelvic disproportion; CS, Cesarean section.

The facility-based CS rates and the frequency of each indication for CS were calculated using descriptive statistics, along with an in-depth analysis of decision-making quality for the most common indications of CS (Figure 2). The proportions of CSs categorized as 1) medically indicated; 2) nonmedically indicated; and 3) unclear if medically indicated were calculated as proportions of the total number of CSs and of each of the most common indications. Following data analysis, 2 meetings were held with obstetricians from the facilities to discuss preliminary findings and ensure contextual interpretation.

Hansen. Disclosing possible nonmedically indicated cesarean sections in 5 high-volume urban maternity units in Tanzania: a criterion-based clinical audit. AJOG Glob Rep 2024.

## Results

During the 3 months in each of the 5 maternity units, a total of 9364 women gave birth, out of which 2949/9364 (31.5%) underwent CSs. Facility-based CS rates ranged from 20.1% to 41.7% (Figure 1). Case-files with a documented CS indication were available for 2674/2949 (90.7%). Among these, 688/2674 (25.7%) were referred from lower-level HF, 790/2674 (29.5%) were nulliparous, and 1157/2674 (43.3%) had a previous CS.

Previous CS was the primary indication for CS (1133/2674, 42.4%), followed by prolonged labor (746/2674, 27.9%), fetal distress (554/2674, 20.7%), hypertensive disorders in pregnancy (216/2674, 8.1%), abnormal presentation (134/2674, 5.0%), breech (84/2674, 3.1%) and other indications (446/2674, 16.7%) (Figure 2, Table S2). More than one indication for CS was recorded in 659/2674 (24.6%) women.

Upon admission, at least 1889/2674 (70.6%) were not yet in active labor (Table 2). A written CS decision note was present in 2364/2674 (88.4%) files and FHR at the time of the decision was recorded in 2149/2674 (80.4%) case-files. At the time of the CS decision, 2194/2674 (82.0%) were in labor (i.e. latent or active phase of labor), and their decision-to-birth interval was within one hour for 190/2194 (8.7%) and more than 3 hours for 889/2194 (40.5%).

**Table 2:** Maternal characteristics, quality of care and clinical decisions during labor, and outcomes

		All 5 maternity units		HF1		HF2		HF3			HF4		HF5	
		n = 2674	%	n = 581	%	n = 397	%	n = 540	%	n = 678		%	n = 478	%
**Admission**	**Maternal age (n = 2674)**													
	Maternal age, Md (IQR)	28 (24-32)		27 (23-31)		28 (24-32)		29 (24-33)		28 (24-33)			29 (24-32)	
	Missing	60	2.2%	35	6.0%	19	4.8%	6	1.1%	0		0.0%	0	0.0%
	**Parity on admission (n = 2674)**													
	0	790	29.5%	173	29.8%	143	36.0%	154	28.5%	199		29.4%	121	25.3%
	1	763	28.5%	172	29.6%	113	28.5%	160	29.6%	177		26.1%	141	29.5%
	≥2	1027	38.4%	200	34.4%	121	30.5%	220	40.7%	302		44.5%	184	38.5%
	Missing	94	3.5%	36	6.2%	20	5.0%	6	1.1%	0		0.0%	32	6.7%
	**Previous CS (n = 2674)**													
	0	1517	56.7%	391	67.3%	233	58.7%	283	52.4%	391		57.7%	219	45.8%
	1	785	29.4%	145	25.0%	116	29.2%	170	31.5%	172		25.4%	182	38.1%
	≥2	345	12.9%	39	6.7%	47	11.8%	82	15.2%	108		15.9%	69	14.4%
	Yes, unknown number	27	1.0%	6	1.0%	1	0.3%	5	0.9%	7		1.0%	8	1.7%
	**Referral case (n = 2674)**													
	Yes	688	25.7%	51	8.8%	7	1.8%	323	59.8%	231		34.1%	76	15.9%
	No	1986	74.3%	530	91.2%	390	98.2%	217	40.2%	447		65.9%	402	84.1%
	**Stage of labor on admission (n = 2674)**^a^													
	Not in labor	632	23.6%	129	22.2%	76	19.1%	223	41.3%	120		17.7%	84	17.6%
	In labor, stage unknown	236	8.8%	99	17.0%	3	0.8%	12	2.2%	49		7.2%	73	15.3%
	Latent phase, first stage (<4 cm)	1257	47.0%	228	39.2%	242	61.0%	195	36.1%	391		57.7%	201	42.1%
	Active phase, first stage (4-<10 cm)	533	19.9%	118	20.3%	74	18.6%	108	20.0%	114		16.8%	119	24.9%
	Second stage	16	0.6%	7	1.2%	2	0.5%	2	0.4%	4		0.6%	1	0.2%
**Decision on CS**	**Doctor's documentation of decision of CS (n = 2674)**													
	Yes	2364	88.4%	561	96.6%	392	98.7%	517	95.7%	643		94.8%	251	52.5%
	No	310	11.6%	20	3.4%	5	1.3%	23	4.3%	35		5.2%	227	47.5%
	**Stage of labor on decision of CS (n = 2674)**													
	Not in labor	466	17.4%	93	16.0%	59	14.9%	167	30.9%	81		11.9%	66	13.8%
	In labor, stage of labor unknown	115	4.3%	22	3.8%	2	0.5%	8	1.5%	18		2.7%	65	13.6%
	Latent phase of labor (<4 cm)	916	34.3%	154	26.5%	142	35.8%	155	28.7%	332		49.0%	133	27.8%
	First stage, active labor (4-<10 cm)	1101	41.2%	288	49.6%	182	45.8%	192	35.6%	236		34.8%	203	42.5%
	Second stage	62	2.3%	20	3.4%	12	3.0%	15	2.8%	10		1.5%	5	1.0%
	Missing	14	0.5%	4	0.7%	0	0.0%	3	0.6%	1		0.1%	6	1.3%
	**Decision-to-birth interval** ***Of women in labor on decision of CS (*n = 2194*)***													
	<1 h	190	8.7%	72	14.9%	35	10.4%	18	4.9%	32		5.4%	33	8.1%
	1-2 h	494	22.5%	160	33.1%	87	25.7%	83	22.4%	104		17.4%	60	14.8%
	2-3 h	386	17.6%	93	19.2%	53	15.7%	77	20.8%	104		17.4%	59	14.5%
	>3 h	889	40.5%	107	22.1%	129	38.2%	154	41.6%	345		57.9%	154	37.9%
	Missing	235	10.7%	52	10.7%	34	10.1%	38	10.3%	11		1.8%	100	24.6%
	**Birth outcome (n = 2783)**													
**Outcomes**	Stillbirth	47	1.7%	5	0.8%	4	1.0%	13	2.2%	21		3.0%	4	0.8%
	Neonatal death before discharge	6	0.2%	1	0.2%	0	0.0%	0	0.0%	5		0.7%	0	0.0%
	Transferred to NHCU, outcome unknown^b^	20	0.7%	1	0.2%	6	1.5%	4	0.7%	9		1.3%	0	0.0%
	Alive on discharge	2618	94.1%	548	91.9%	370	90.2%	552	95.3%	671		94.8%	477	97.3%
	Missing^c^	92	3.3%	41	6.9%	30	7.3%	10	1.7%	2		0.3%	9	1.8%
	**Apgar after 5 min (n = 2783)**													
	0-2	48	1.7%	5	0.8%	4	1.0%	13	2.2%	22		3.1%	4	0.8%
	3-7	40	1.4%	3	0.5%	1	0.2%	11	1.9%	14		2.0%	11	2.2%
	8-10	2601	93.5%	547	91.8%	374	91.2%	544	94.0%	670		94.6%	466	95.1%
	Missing	94	3.4%	41	6.9%	31	7.6%	11	1.9%	2		0.3%	9	1.8%
	**Birth weight (n = 2783)**													
	<2500 g	306	11.0%	29	4.9%	29	7.1%	106	18.3%	91		12.9%	51	10.4%
	≥2500 g	2395	86.1%	525	88.1%	361	88.0%	466	80.5%	616		87.0%	427	87.1%
	Missing	82	2.9%	42	7.0%	20	4.9%	7	1.2%	1		0.1%	12	2.4%
	**Maternal outcome (n = 2674)**													
	Death	1	0.0%	0	0.0%	0	0.0%	1	0.2%	0		0.0%	0	0.0%
	Alive	2673	100.0%	581	100.0%	397	100.0%	539	99.8%	678		100.0%	478	100.0%
	**Maternal complications (n = 2674)**													
	No	2590	96.9%	581	100.0%	379	95.5%	507	93.9%	660		97.3%	463	96.9%
	Infection/sepsis	7	0.3%	0	0.0%	4	1.0%	1	0.2%	0		0.0%	2	0.4%
	Postpartum hemorrhage^d^	51	1.9%	0	0.0%	11	2.8%	22	4.1%	9		1.3%	9	1.9%
	Re-surgery	2	0.1%	0	0.0%	0	0.0%	1	0.2%	1		0.1%	0	0.0%
	Hysterectomy	10	0.4%	0	0.0%	1	0.3%	2	0.4%	3		0.4%	4	0.8%
	Other	14	0.5%	0	0.0%	2	0.5%	7	1.3%	5		0.7%	0	0.0%

CS, Cesarean section; HF, Health facility; IQR, Interquartile range; Md, Median; NHCU, Neonatal high care unit

a If unknown status of labor on admission, women were defined to be in labor.

b Newborns were transferred to neonatal high care unit and outcome could not be followed up.

c Newborns with unknown Apgar after 5 minutes, with no documentation of being referred to neonatal high care unit.

d Defined by blood loss >1000 mL or documentation of severe anemia or postpartum hemorrhage.

One maternal death following CS was reported and 84/2674 (3.1%) had documented maternal complications postpartum before discharge (e.g., postpartum hemorrhage). The median time between birth and discharge was 2 days (IQR 1-2, missing n = 149). In total, 2601/2783 (93.5%) newborns had an Apgar 8-10 after 5 minutes. Among newborns, 47/2783 (1.7%) were stillbirths, of which 25/47 (53.2%) had a positive FHR recorded at the time of CS decision. Another 118/2783 (4.2%) were transferred to a neonatal high care unit, of which 6/118 (5.1%) died and 20/118 (16.9%) could not be traced.

### Estimation of nonmedically indicated CSs

In total, 1010/2674 (37.8%) of all CSs were audited as being medically indicated at the time when decided upon, 1061/2674 (39.7%) as nonmedically indicated, and 603/2674 (22.6%) as unclear if medically indicated. The most common indications are described below, with audit criteria detailed in Table 1 and further details available in Figure 2.

Among women undergoing CS due to a previous CS (1133/2674, 42.4%), 473/1133 (41.7%) were categorized as “medically indicated CS”, 526/1133 (46.4%) were categorized as “nonmedically indicated CS” (no trial of labor after only one previous CS (TOLAC)), and 134/1133 (11.8%) were categorized as unclear. Among women with a previous CS, 352/1133 (31.1%) were not in labor on admission while 552/1133 (48.7%) were in the latent phase of labor and 139/1133 (12.3%) in the first stage of active labour.

Among women undergoing CS due to prolonged labor (746/2674, 27.9%), 213/746 (28.6%) were categorized as “medically indicated CS,” 243/746 (32.6%) were categorized as “nonmedically indicated CS” (prolonged labor reported without actual slow labor progress), and 290/746 (38.9%) were categorized as unclear.

Among women undergoing CS due to fetal distress (554/2674, 20.7%), 19/554 (3.4%) were categorized as “medically indicated CS,” 211/554 (38.1%) were categorized as “nonmedically indicated CS” (normal FHR ((120-160 bpm). recorded at time of decision), and 324/554 (58.5%) were categorized as unclear. For CSs performed due to fetal distress, 429/564 (76.1%) babies were born more than 1 hour after the CS decision and scored Apgar 8-10 after 5 minutes. Among all fetal distress cases with a normal FHR upon CS decision (n = 270), 151/270 (55.9%) had meconium-stained liquor and 34/270 (12.6%) reported reduced fetal movements (Table S3).

## Structured discussion

At the 5 urban maternity hospitals in Tanzania, our study revealed a CS rate of 31.5%, which is significantly higher than both the national average and the average for DSM.^16^ Our case-file reviews of management preceding 2674 CSs disclosed that 39.7% of the surgeries appeared nonmedically indicated upon time of decision, main reasons being absence of TOLAC and nonsubstantiated diagnoses of prolonged labor and fetal distress. This illustrates the vicious cycle underlying the CS increase: prolonged labor and fetal distress are often over-diagnosed during first birth, and the first CS almost always leads to repeated CSs.^3^^,^^4^^,^^5^^,^^26^^,^^33^

These findings are consistent with trends in other low- and middle-income countries’ urban settings (e.g. Kenya, Malawi), where rising CS rates coincide with a growing urban disadvantage in maternal and perinatal health.^10^^,^^34^, ^35^ To break the vicious circle of repeated CS, preventing the first CS is essential. We discuss management of prolonged labor in separate papers,^32^^,^^36^ and focus here on fetal distress and previous CS. Health providers often diagnosed fetal distress by evaluating meconium-stained fluid and reduced fetal movements, despite these signs having poor association with fetal distress when FHR is normal (Table S3).^37^^,^^38^ Accordingly, 94% (529/564) of babies born by CS due to fetal distress had Apgar score ≥8 after 5 minutes. Notably, an audit of all perinatal deaths at the study hospitals in 2020 found a perinatal mortality rate of 38/1000 total births, and CS did not cause a significantly increased risk.^39^ In light of our results, this highlights a dangerous co-existence of under- and overtreatment in these facilities, where some women undergo CS which is not medically indicated, while others receive suboptimal care during vaginal birth. Similar overdiagnosis of fetal distress has been observed in Nepal and Burkina Faso, and timely detection of fetuses at risk through intermittent auscultation is particularly challenging in crowded understaffed maternity units.^4^^,^^40^

Furthermore, delays in accessing the single operating theatre may lead to earlier-than-indicated CS decisions: Only 8.7% of CSs for women in labor had a decision-to-birth interval of less than one hour. These challenges strongly highlight the urgent need for more timely surveillance during vaginal birth, including better means to assess fetal well-being, and ensure emergency CSs without delays.^41^, ^42^, ^43^

In high-income countries, the risk of uterine rupture following TOLAC is low (<0.005%), supporting its global recommendation.^44^ However, evidence on TOLAC safety in low-resource settings is limited. An observational study from Senegal and Mali involved 9712 women and reported a 45% likelihood of successful TOLAC, while risks of mortality and uterine rupture were low.^45^ Meanwhile, maternal death reviews from other low-resource settings, including Ethiopia and Pakistan, report concerns about TOLAC, a fear shared by obstetricians at the study facilities.^46^, ^47^, ^48^ However, interpretation of these maternal death reviews among women undergoing TOLAC is complicated by limited comparison to the short- and long-term risks of CS. Furthermore, research is scarce on women's preferences for birth after CS. Notably, qualitative findings from one of our study hospitals revealed that women with a previous CS often intentionally arrive late at the facility during childbirth to increase their chances of attempting TOLAC.^46^

### Clinical implications

Our findings suggest that CS decisions were often made too soon. As reported in a study from DSM, this could be because of defensive decision making driven by fear of unsafe vaginal births and blame.^49^ This aligns with conversations held with the staff at the five hospitals. While short-term outcomes after CS were reassuring, research is needed to unfold the long-term consequences in this urban, low-resource setting with a fertility rate of 3.6.^16^ Moreover, considering that these study facilities suffer from high and stagnating burdens of perinatal mortality, this defensive, individually-based decision-making on CS neglects the collective need for resources and the costing cascades that strain already scarce resources.^39^^,^^50^, ^51^, ^52^ Considering the staff shortages, high rates of CSs inevitably divert attention from other laboring women during vaginal birth, increasing their obstetric risks.^31^ This further emphasizes the need for a collective, structured, and rights-based approach within urban resource-constrained healthcare systems to ensure best possible and timely care for all.^12^ To move beyond the current defensive CS decision-making, healthcare providers at the study sites emphasized the importance of securing continuous support with timely surveillance during vaginal birth and shortening the decision-to-delivery interval for emergency CS. In addition, context-specific clinical guidelines on timely CS decisions, mandatory second opinions, shared decision-making, and training in prolonged labor, TOLAC and assisted vaginal birth are crucial strategies to reduce CS.^40^^,^^53^, ^54^, ^55^, ^56^

### Research implications

There is an urgent need for context-specific decision-support tools on *when* to perform CSs, particularly concerning prolonged labor in nulliparous women and fetal distress. Also, with the rapid CS increase, research on safe TOLAC and long-term CS outcomes in resource-constrained, high-volume maternity units is crucial to dispel potentially unnecessary fears of TOLAC risks and maximize benefits.^57^

### Strengths and limitations

This study provides insight into the challenges of CS decision-making in low-resource urban maternity units. Notably, compared to the Robson classification, such a detailed audit is essential for formulating actions for improvement.^58^ Additional strengths include a high proportion of assessed CS files (90.7%; Figure 1) and double-entry of 30.3% of cases. A prior data validation study assessed the completeness and accuracy of case-file data by clinical observations, including FHR (accuracy: 73.5%, 85.5%) and vaginal examination (accuracy: 76.0%, 90.8%) (unpublished observations). While findings may not be universally applicable, their consistency across the facilities and similarity to other low-resource settings, support their broader relevance.^4^^,^^20^^,^^27^^,^^40^^,^^59^

Key limitations include the retrospective design and the "not documented, not performed" principle as applied in most criterion-based audits.^20^^,^^31^ Understanding CS decisions involves multiple factors, some of which were not documented in the case-files (e.g., the woman's preferences). While this warrants further research, we ensured contextual accuracy by discussing preliminary findings with obstetricians from the study facilities. Moreover, some women undergoing a nonmedically indicated CS would have needed a CS later during vaginal birth. This would e.g. be the case for 10-40% of women undergoing TOLAC.^60^, ^61^, ^62^ Lastly, complications arising after discharge were not included, and the number of women undergoing vaginal birth who would have benefitted from a CS remains uncertain.

## Conclusions

We found that 39.7% of 2674 CSs appeared nonmedically indicated at time of decision, main indications being one previous CS, prolonged labor and fetal distress. To promote judicious decision-making on mode of birth, regular CS audits must be coupled with investments to create conducive maternity units providing safe vaginal birth with the option of timely emergency CS. Policy and practice must ensure urban healthcare systems that cover the entire population through a collective-oriented approach in clinical decision-making. This shift is critical, as the demand for facility births in many cities is increasing faster than the expansion of health systems.

## CRediT authorship contribution statement

**Sarah Hansen:** Writing – review & editing, Writing – original draft, Visualization, Validation, Methodology, Funding acquisition, Formal analysis, Data curation, Conceptualization. **Monica Lauridsen Kujabi:** Writing – review & editing, Validation, Supervision, Project administration, Methodology, Funding acquisition, Formal analysis, Data curation, Conceptualization. **Rikke Damkjær Maimburg:** Writing – review & editing, Supervision, Methodology, Funding acquisition. **Anna Macha:** Writing – review & editing, Data curation. **Luzango Maembe:** Writing – review & editing, Validation, Methodology. **Idrissa Kabanda:** Writing – review & editing, Validation, Methodology. **Manyanga Hudson:** Writing – review & editing, Validation, Methodology. **Rukia Juma Msumi:** Writing – review & editing, Validation, Methodology. **Mtingele Sangalala:** Writing – review & editing, Validation, Methodology. **Natasha Housseine:** Writing – review & editing, Validation, Methodology, Conceptualization. **Brenda Sequeira D'mello:** Writing – review & editing, Validation, Methodology, Conceptualization. **Kidanto Hussein:** Writing – review & editing, Methodology, Conceptualization. **Thomas van den Akker:** Writing – review & editing, Methodology, Conceptualization. **Dan Wolf Meyrowitsch:** Writing – review & editing, Funding acquisition, Conceptualization. **Nanna Maaløe:** Writing – review & editing, Supervision, Project administration, Methodology, Funding acquisition, Formal analysis, Data curation, Conceptualization.

## Appendix Supplementary materials

Image, application 1

## Footnotes

Supplementary material associated with this article can be found, in the online version at doi:10.1016/j.xagr.2024.100437.
