# Supplementary material for: Disclosing possible nonmedically indicated cesarean sections in 5 high-volume urban maternity units in Tanzania: a criterion-based clinical audit
Source: AJOG Glob Rep. 2024 Dec 21;5(1):100437. doi: 10.1016/j.xagr.2024.100437 (PMC11786107; doi:10.1016/j.xagr.2024.100437)
Supplement: Supplementary file 1 [file mmc1.docx]

**Supplementary tables and figures**

| **Figure S1: Map of the five included maternity units in Dar es Salaam, Tanzania. *Reused under the terms of the Creative Commons CC BY license*[22].** |
| --- |
| **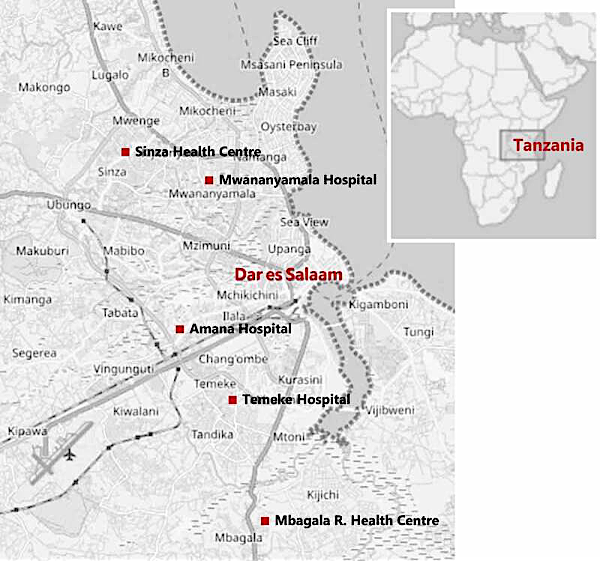** |

| **Table S2: Distribution of indications for cesarean section illustrating the proportion of each indication. Each case may have more than one indication.** | | | | | | | | | | | | | | |
| --- | --- | --- | --- | --- | --- | --- | --- | --- | --- | --- | --- | --- | --- | --- |
|  | **All five maternity units** | | | | **HF1** | | **HF2** | | **HF3** | | **HF4** | | **HF5** | |
|  | **Distribution of indication(s)** | | **Proportion of each indication** | | | | | | | | | | | |
|  | **n=2674** | **%** | **n=2674** | **%** | **n=581** | **%** | **n=397** | **%** | **n=540** | **%** | **n=678** | **%** | **n=478** | **%** |
| Previous CS | 964 | 36.1% | 1133 | 42.4% | 179 | 30.8% | 164 | 41.3% | 253 | 46.9% | 280 | 41.3% | 257 | 53.8% |
| Prolonged labor^a^ | 467 | 17.5% | 746 | 27.9% | 258 | 44.4% | 127 | 32.0% | 110 | 20.4% | 128 | 18.9% | 123 | 25.7% |
| Fetal distress | 268 | 10.0% | 554 | 20.7% | 97 | 16.7% | 67 | 16.9% | 99 | 18.3% | 201 | 29.6% | 90 | 18.8% |
| Hypertensive disorders in pregnancy | 38 | 1.4% | 216 | 8.1% | 4 | 0.7% | 5 | 1.3% | 115 | 21.3% | 67 | 9.9% | 25 | 5.2% |
| Abnormal presentation | 73 | 2.7% | 134 | 5.0% | 27 | 4.6% | 14 | 3.5% | 32 | 5.9% | 40 | 5.9% | 21 | 4.4% |
| Breech | 42 | 1.6% | 84 | 3.1% | 24 | 4.1% | 12 | 3.0% | 15 | 2.8% | 17 | 2.5% | 16 | 3.3% |
| Other indication(s)^b^ | 163 | 6.1% | 446 | 16.7% | 86 | 14.8% | 70 | 17.6% | 111 | 20.6% | 121 | 17.8% | 58 | 12.1% |
| $\geq$2 indications | 659 | 24.6% |  |  |  |  |  |  |  |  |  |  |  |  |
| ^a^ Indications included: Prolonged labor, cephalopelvic disproportion, poor progress of labor, cervical arrest, failure of augmentation, obstructed labor, big baby.  ^b^ Includes the following:  Defined as medically indicated: Cord prolapse with pulsating cord n=38, placenta abruptio n=23, placenta praevia n=23, uterine rupture n=7, retained second twin n=5, vacuum failure n=5, previous reconstructive vaginal surgery n=4, cervical stenosis n=3, obstetric tumor n=3, cervical/uterine prolapse n=2, Bartholin's edema n=1, hip dislocation in previous pregnancy n=1, pelvic injury n=1, psychosis n=1, transverse vaginal septum n=1.  Defined as nonmedically indicated: Bad obstetric history n=73, oligohydramnios (normal FHR) n=38, placenta calcification n=26, post date n=24, premature rupture of membranes n=20, long interpregnancy interval n=19, other rarely used indications n=49 (e.g. elderly maternal age, twins).  Defined as unclear if medically indicated: Failure of induction n=49, antepartum bleeding (no placenta abruptio, no placenta praevia) n=36, other rarely used indications n=51 (e.g. anemia, myoma). All indications are presented in audit criteria (Table 1).  CS, Cesarean section; FHR, Fetal heart rate, HF, Health facility | | | | | | | | | | | | | | |

| **Table S3: Possible characteristics to understand the complexity of why women undergo CS due to fetal distress with a normal FHR (120-160 bpm) on decision of CS (n=270)^a^. Each case may have more other indications or characteristics.** | | |
| --- | --- | --- |
|  | **n=270** | **%** |
| **Other characteristics on decision of CS** |  |  |
| Meconium-stained liquor | 151 | 55.9% |
| Reduced fetal movement | 34 | 12.6% |
| Irregular rhythm | 15 | 5.6% |
| **Other indications for CS** |  |  |
| Severe hypertensive disorders in pregnancy | 24 | 8.9% |
| Bad obstetric history | 9 | 3.3% |
| Oligohydramnios | 6 | 2.2% |
| Failure of induction | 4 | 1.5% |
| Rarely used indications (e.g. anemia, polyhydramnios, post date) | 13 | 4.8% |
| Another absolute indication^b^ | 39 | 14.4% |
| ^a^ Outcome of the newborns following CS due to fetal distress with a normal FHR on decision (n=275): 4/275 (1.5%) had Apgar 0-2 after five minutes, 23/275 (8.4%) had Apgar 3-7 after five minutes, 262/275 (95.3%) had Apgar 8-10 after five minutes and 5/275 (1.8%) had unknown Apgar after five minutes.  ^b^ Other absolute indications for CS: Prolonged labor n=22, two previous CS or one previous CS with failed trial of labor n=4, breech with failed trial of labor n=2 and other indications (e.g. abnormal presentation, cord prolapse, placenta abruptio) n=11.  CS, Cesarean section; FHR, Fetal heart rate | | |
